# Genomic and Mitonuclear Patterns of Divergence Among Recently Diverged White‐Crowned Sparrow Subspecies

**DOI:** 10.1002/ece3.73651

**Published:** 2026-05-14

**Authors:** Patricia B. Osagie, Juan Enciso‐Romero, Theresa M. Burg

**Affiliations:** ^1^ Department of Biological Sciences University of Lethbridge Lethbridge Alberta Canada

**Keywords:** divergence, outlier SNPs, population, white‐crowned sparrow, Z chromosome

## Abstract

Genetic divergence and subsequent speciation are possible within the range and habitat of a population. White‐crowned sparrows are comprised of five subspecies that thrive in a wide range of habitats, latitudes, and elevations across North America, suggesting the ability for local adaptation and rapid divergence. We assessed genome‐wide genetic differentiation in four populations of white‐crowned sparrow ( *Zonotrichia leucophrys gambelii* , *Z. l. oriantha* (northern and southern), and *Z. l. pugetensis*) and compared patterns of genetic structure using outlier single nucleotide polymorphisms (SNPs), including mitochondrial (255 SNPs) and Z chromosome (580 SNPs) loci. When these four populations are assessed using the full SNP dataset, three genetic clusters are identified: *pugetensis*, southern *oriantha*, and northern *oriantha* grouped with *gambelii*. However, four genetic clusters are supported by the Z chromosome outlier SNPs corresponding to three subspecies and a north–south split in *Z. l. oriantha*. Our analyses pointed to consistent divergence of *Zonotrichia leucophrys pugetensis* from other populations and mitochondrial SNPs showed overlap of southern *Z. l. oriantha* and *Z. l. gambelii*. Interestingly, northern *Z. l. oriantha* populations are more genetically similar to *Z. l. gambelii* as opposed to its sister population, the southern *Z. l. oriantha*. We found a region on the Z chromosome with highly elevated *F*_ST_ among all the populations. The genetic clusters identified in our study may be suggestive of evolutionary events such as selection on the Z chromosome, and this may be driving divergence in white‐crowned sparrows.

## Graphical abstract

The study investigates genetic divergence among white‐crowned sparrow subspecies across North America, revealing three to four distinct genetic clusters based on genome‐wide SNP analysis, particularly highlighting divergence in the Z chromosome. Notably, *Z. l. pugetensis* consistently diverges from other populations, and a north–south split within *Z. l. oriantha* suggests localized adaptation and potential speciation events. Elevated genetic differentiation on the Z chromosome may indicate selection‐driven evolutionary processes contributing to this divergence.

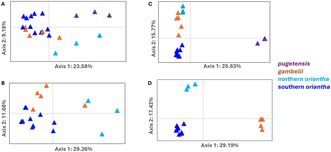

## 1 Introduction

Advances in genomic sequencing techniques have opened the door to a wide range of genetic studies including population structure and signatures of selection (Andrews et al. 2016; Jeffries et al. 2016; Hodel et al. 2017; Morgan et al. 2017; Bohling et al. 2019). Genome‐scale data are not limited to just neutral genetic variation; they also allow us to detect loci under selection (Hedrick 1999; Primmer 2009) and identify potential loci of interest for local adaptation (Steiner et al. 2013). Further, genomics is useful in studying how populations adapt and evolve in response to local environmental conditions. For example, genome‐wide scans have identified evidence of selection and differentiation resulting from local adaptation to salinity in Baltic Sea herring (Guo et al. 2016); and urban land use in both white‐footed mouse (Munshi‐South et al. 2016) and red‐tailed bumblebee (Theodorou et al. 2021).

Species with large geographic distributions often inhabit diverse habitat types and encounter a range of environmental conditions and physical barriers to dispersal. Claramunt et al. (2025) found populations within microhabitats may experience isolation. Similarly, high latitude species experienced large range shifts during the Pleistocene glaciations, adapting to lower latitudes and elevations where they survive in refugia and evolved in isolation, sometimes resulting in morphological, behavioral, and genetic differences (Weir and Schluter 2004; Pielou 2008; Hewitt 2000).

**FIGURE 1:**
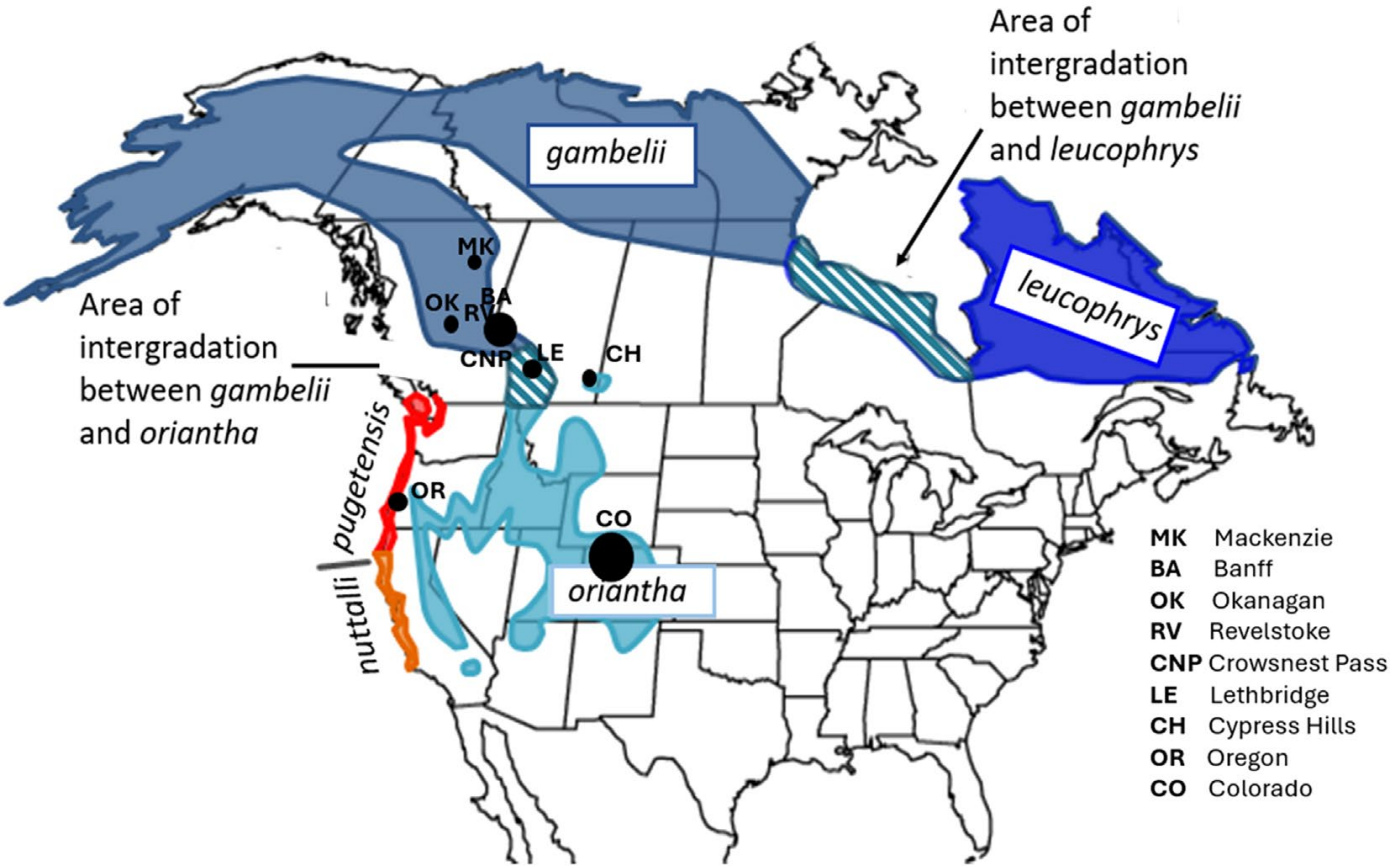
Breeding season range map of white‐crowned sparrow subspecies indicating distribution and intergradation. The two Pacific groups: *Z. l. nuttalli* (orange) and *pugetensis* (red) have an overlapping range in northern California. Two other areas of overlap are shown on the map with crosshatching between *Z. l. oriantha* (teal), *Z. l. gambelii* (blue‐gray) and *Z. l. leucophrys* (navy blue). From Welke et al. (2021). Black circles represent sampling locations, and the circle size corresponds to the number of samples.

The white‐crowned sparrow, *Zonotrichia leucophrys* , has a wide distribution and exhibits phenotypic diversity across its range (Chilton et al. 1990; Dunn et al. 1995; Morton 2002) suggesting adaptation may have resulted from isolation in different refugia during the last glacial maximum (LGM) (Taylor et al. 2021; Welke et al. 2021). The present‐day populations of white‐crowned sparrows are found at high latitudes or high elevations in Canada and the US (Figure 1): *Z. l. gambelii* in northern and western Canada and Alaska; *Z. l. leucophrys* in northeastern Canada; *Z. l. oriantha* in the southern Rocky Mountains and Sierra Nevada; *Z. l. nuttalli* and *Z. l. pugetensis* west of the Cascade Mountain Range (Rand 1948). The five subspecies have several distinguishing features: *Z. l. nuttalli* and *Z. l. pugetensis*, the two subspecies found in scrub habitat along a narrow strip of the Pacific Coast (Figure 1), have pale lores, a yellow beak, drabber underparts, shorter wings and duller head stripes. *Z. l. gambelii, Z. l. oriantha* and *Z. l. leucophrys* are grouped as boreal and montane birds. They are generally grayish below with deep reddish, and pale gray stripes on the back. *Z. l. gambelii* from the western boreal forest has white/gray lores and an orange bill whereas *Z. l. oriantha* from the interior west/Rocky Mountains has black lores with a pink bill. *Z. l. leucophrys* from the eastern boreal is very similar to *Z. l. oriantha* and may be difficult to distinguish in the hand though they have distinct, non‐overlapping breeding areas (Banks 1964). In addition to morphology, there is ecological and behavioral variation among white‐crowned sparrow subspecies. *Z. l. nuttalli* is non‐migratory (Hafner and Petersen 1985); and the other four subspecies are migratory. Both *Z. l. oriantha* and *Z. l. gambelii* have strong fidelity to their wintering sites in the southwestern US and northern Mexico and breeding sites in the north (Morton 2002).

Several studies using mitochondrial, microsatellite and single nucleotide polymorphism datasets have pointed to the divergence of white‐crowned sparrow subspecies with varying degrees of support. Mitochondrial DNA show the presence of some unique haplotypes within *Z. l. nuttalli* (Taylor et al. 2021); however, there is some overlap with the other subspecies and the other four subspecies show no differentiation (Taylor et al. 2021; Welke et al. 2021). In contrast, using nuclear markers there is clear separation of western subspecies *Z. l. pugetensis* and *Z. l. nuttalli* from the other subspecies; however, among *Z. l. gambelii, Z. l. oriantha* and *Z. l. leucophrys* differentiation is either weak or patterns are inconclusive (Taylor et al. 2021; Welke et al. 2021). Furthermore, Welke et al. (2021) found that population structure in the Canadian Rocky Mountains between *Z. l. gambelii* and *Z. l. oriantha* corresponded to habitat differences, not subspecies. One reason for the inconsistent patterns of genetic differentiation with the use of mitochondrial and nuclear markers could be incomplete lineage sorting from recent separation or hybridization in areas of range overlap especially the three eastern subspecies (Figure 1).

Whole genome sequencing provides a more comprehensive picture of divergence patterns and can allow us to focus on specific parts of the genome that may differ among closely related groups of individuals (Taylor et al. 2021; Wang et al. 2021; Oyler‐McCance et al. 2015). Sex chromosomes, especially the Z chromosome, show more rapid divergence than autosomes (Hooper and Price 2017; Irwin 2018), and some song and plumage coloration genes are found on the Z chromosome (Choe and Jarvis 2021; Cumer et al. 2024), that could contribute to reproductive isolation. Given the low levels of differentiation found among some of the subspecies, the higher resolution whole genome sequencing may be better able to resolve the differences. In addition, subspecies of white‐crowned sparrows show differences in lore coloration (gray in *gambelii* and black in *pugetensis*), bill coloration (orange in *gambelii*, dark reddish pink in *oriantha*, and dull yellow in *pugetensis*), call note characteristics, and migratory behavior (Dunn et al. 1995; Banks 1964; Hafner and Petersen 1985), we hypothesize that potential loci under selection may have roles in pigmentation, auditory processes, and skeletal muscle development. Our study used low coverage whole genome sequencing (lcWGS) data and outlier analyses to study three of the recently diverged subspecies, *Z. l. gambelii*, *Z. l. oriantha* and *Z. l. pugetensis*, and examine genes that may be under selection. Based on results from previous studies, we predict the presence of four genetic groups corresponding to the three subspecies with additional differences between northern and southern populations of *oriantha* and that these differences will correspond to genes under selection.

## 2 Methods

### 2.1 Sample Collection

A total of thirty‐nine samples were initially collected from sites across North America (Figure 1). *Z. l. oriantha* (*n* = 6), *Z. l. gambelii* (*n* = 17) and one unknown sample were from both western and southern North America (Crowsnest Pass, Cypress Hills, Lethbridge, Banff, Mackenzie, Revelstoke, and Okanagan), with additional southern *Z. l. oriantha* (*n* = 12) from Gunnison County, Colorado, and *Z. l. pugetensis* (*n* = 3) from Oregon (Table 1). Samples were selected to avoid effects of habitat on variation for *Z. l. gambelii* and *Z. l. oriantha* that co‐occur in both alpine coniferous and riparian deciduous habitat types as Welke et al. (2021) found genetic differences among birds from the two habitats. Only samples from riparian deciduous habitat for these two subspecies were included in the analyses.

**TABLE 1:** Sample location and size where study samples were collected.

Sample location	Subspecies (# of sample)	Latitude	Longitude
MA (Mackenzie)	*gambelii* (3)	55.31	−123.12
BA (Banff)	*gambelii* (3), northern *oriantha* (1)	51.17	−115.56
OK (Okanagan)	*gambelii* (3)	49.34	−119.57
RV (Revelstoke)	*gambelii* (2), *unknown* (1)	50.99	−118.19
CNP (Crowsnest Pass)	northern *oriantha* (3), *gambelii* (1)	49.73	−114.60
LE (Lethbridge)	*gambelii* (4)	49.69	−112.83
CH (Cypress Hills)	northern *oriantha* (2), *gambelii* (1)	49.56	−110.13
OR (Oregon)	*pugetensis* (3)	45.18	−123.47
CO (Colorado)	Southern *oriantha* (12)	38.98	−107

Sample collection occurred during the breeding season between late May and July (2017–2021) using 12 m mist net and bird song playback. Up to 50 μL of blood was collected from the brachial vein of each bird, or a tail feather was collected. Blood samples were placed in 99% ethanol and stored at −20°C upon return to the lab; feather samples were placed in individual tubes and preserved at −20°C. Before being released, birds were photographed in case we needed to look at plumage, and banded with a numbered metal band to avoid resampling.

### 2.2 DNA Extraction, Library Preparation, and Sequencing

The DNA extraction followed a similar protocol of Aljanabi and Martinez (1997) with a salt extraction to obtain high yield DNA. We modified the DNA protocol by adding glycol blue to our supernatant after the precipitation step to allow enhanced visibility of the DNA pellet. We also did two ethanol washes to ensure all of the salts were removed. For low coverage whole genome sequencing, a shotgun PCR free library was prepared with 8 bp unique barcode tagged to each sample. Sequencing was done at Genome Quebec on a paired‐end run on Illumina Novaseq 6000 S4 PE 150.

### 2.3 LcWGS Pipeline

Our samples were sequenced at 6.2 to 8.9× depth of coverage. BWA‐MEM implemented in BWA v0.7.15 (Li 2013) was used to align the reads to the annotated genome of zebra finch *Taeniopygia guttata* (Warren et al. 2010; Rhie et al. 2021; reference genome version bTaeGut2.pat.W.v2, GenBank accession number GCA_008822105.2). After filtering out samples with low quality sequences or excessive missing data, 33 samples were retained. We used picard tools v.2.26.3 (http://broadinstitute.github.io/picard/) to remove PCR duplicates and unmapped reads. Reads were then left‐realigned around indels using the bamleftalign command from freebayes v1.3.6 (Garrison and Marth 2012), and we clipped overlapping reads using the clipOverlap function in bamUtils (Jun et al. 2015). We estimated genotype likelihoods using samtools (Li et al. 2009) and bcftools (Li 2011; Danecek et al. 2021) via the mpileup function. We used the bcftools call function for SNP and genotype calling with QUAL > 20. The output of this pipeline was used in pairwise *F*_ST_ and DAPC based structure analysis.

### 2.4 Genome‐Wide *F*_ST_ Scan and Outlier Loci

We ran sliding window *F*_ST_ scans to identify outliers. We used ANGSD v0.933 (Korneliussen et al. 2014) to first estimate the folded allele frequency spectrum using the GATK genotype likelihood model, with minimum base and mapping qualities of 20, and then we ran the realSFS program and the Bhatia *F*_ST_ estimator (Bhatia et al. 2013) using a window of size 25,000 and a step size of 10,000. To identify outlier SNPs and loci that are putatively under selection, we used a quantile‐based outlier analysis implemented in R, setting a quantile threshold of 0.999 over the sliding window *F*_ST_ estimates from ANGSD (Korneliussen et al. 2014). Candidate loci are defined as those falling in the extreme tails of the empirical genome‐wide distribution of differentiation statistics. This empirical framework minimizes model assumptions and is more robust to complex or non‐equilibrium demographic histories under which Bayesian *F*_ST_‐outlier methods such as BayeScan can exhibit elevated false‐positive rates. Quantile‐based methods are also computationally efficient and transparent, making them well suited for exploratory genome scans in large SNP datasets (Lotterhos and Whitlock 2014; Foll 2010).

### 2.5 Evidence of Genetic Structure and Differentiation (Whole Genome vs. Mitochondrial vs. Z Chromosome)

Datasets with outlier SNPs on the Z chromosome or mitochondrial genome were used to test for genetic structure for both DAPC (Jombart et al. 2010) in Adegenet 2.0.0 implemented in RStudio v.1.3.1093 (R Core Team 2020) and PCoA implemented in GenAlEx 6.51b2 (Peakall and Smouse 2012). Our DAPC program first transformed individual multilocus genotypes into principal components (PCs) to reduce dimensionality; we retained PCs based on trade‐offs between explanatory power and overfitting following recommendations in Adegenet. We used the retained PCs as input for discriminant analysis. We inferred the number of genetic clusters (K) using the *find.clusters* function. We further inferred population structure with the whole dataset using ANGSD v0.933 (Korneliussen et al. 2014) to get major and minor alleles, estimate allele frequencies, and retain sites with minor allele frequencies (MAF) > 0.05. Site allele frequencies and population genetic were inferred from genotype likelihoods across individuals, and this formed our input file for the PCA and NGSAdmix analyses. For PCA, we used the sampling approach implemented in ANGSD v0.933 to calculate the genetic covariance matrix for the full dataset. We calculated genotype likelihoods with the samtools model (−GL 1) using a cutoff *p*‐value for SNPs of 2 × 10^−6^, a minimum base quality of 20 and mapping quality of 30 Phred score units, setting a minimum of 29 individuals to support genotype likelihood inference, removing low quality reads, re‐calculating base alignment quality (Li 2011), and adjusting mapping quality for excessive mismatches. We then computed the spectral decomposition in base R (R Core Team 2020, version 1.3.1093). We estimated individual admixture proportions in NGSadmix (Skotte et al. 2013) using genotype likelihoods in Beagle format pre‐computed in ANGSD v0.933 (Korneliussen et al. 2014) and the same filtering settings used for PCA. We varied the assumed number of genetic clusters (K) from two to four and ran ten iterations for each K using different starting seeds. We then used CLUMPAK (Kopelman et al. 2015) to summarize results across each K and plot our results.

To assess regions of the genome contributing to subspecies differentiation, the complete genome output file from the samtools and bcftools pipeline was analyzed for patterns of genetic differentiation. As a previous study showed two genetically distinct groups within *oriantha* corresponding to northern and southern groups (Welke et al. 2021), we also categorized our *oriantha* samples into northern (BC and AB) and southern (CO) groups. VCF tools v0.1.13 per‐site functionality vcftools ‐‐vcf sample.vcf ‐‐weir‐fst‐pop1.txt ‐‐weir‐fst‐pop2.txt (Danecek et al. 2011) was used to compute Weir and Cockerham *F*_ST_ values for all pairwise combinations among subspecies and groups: *Z. l. gambelii*, *Z. l. pugetensis*; northern *Z. l. oriantha* and southern *Z. l. oriantha* to give an overview of chromosomes that may be contributing to divergence and have high values of *F*_ST_. Both the Z chromosome and mtDNA show higher rates of evolution and different modes of inheritance relative to autosomal chromosomes (Hooper and Price 2017; Irwin 2018; McCallum et al. 2024). To explore the degree of genetic differentiation between the subspecies, we calculated pairwise *F*_ST_ in Arlequin v3.5.2 (Excoffier and Lischer 2010) using the whole genome dataset. To estimate chromosome‐level differentiation and map the distribution of genetic variation, we estimated nucleotide diversity using VCFtools v0.1.13 (Danecek et al. 2011).

### 2.6 Gene Identification

We identified genes near outlier loci using the NCBI gene viewer (Brown et al. 2015). Genomic regions surrounding outlier loci were annotated using the NCBI Gene database and NCBI Genome Data Viewer. For each outlier locus, we defined chromosomal coordinates based on the zebra finch reference genome assembly. We considered genes to be proximal to an outlier locus if they were located within ±500 kb upstream or downstream. We determined the genes' function using gene ontology program, Database for Annotation, Visualization, and Integrated Discovery (DAVID) (Sherman et al. 2022). We further explored the functions and the possible roles of these genes in our populations based on supplemental literature searches.

## 3 Results

### 3.1 Population Genetic Structure in the *Zonotrichia leucophrys* Subspecies: Pugetensis, Gambelii and Oriantha

**FIGURE 2:**
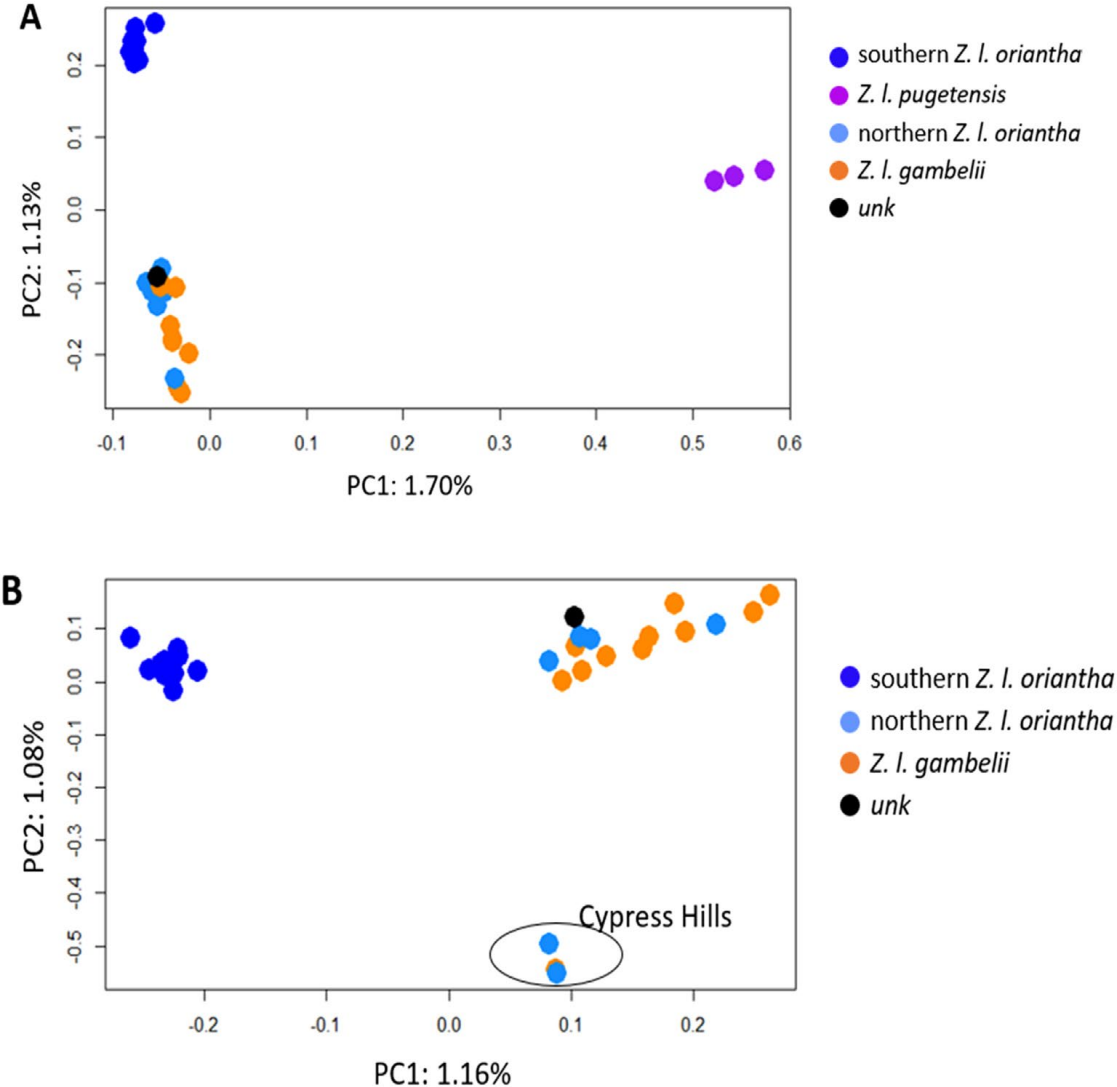
(A) PCA plot of *Z. leucophrys* using lcWGS data shows three genetic clusters: *Pugetensis* and southern *oriantha* each formed a distinct cluster and northern *oriantha* grouped with *gambelii* to form a third cluster. PC1 explains 1.7% of the variation and separates *pugetensis* from the other subspecies. PC2 explains 1.13% of the variation and separates the three groups. (B) PCA run without pugetensis. PC1 contains 1.16% of the variation and separates southern *oriantha* from the northern *oriantha* and gambelii while PC2 (1.08% variation) separates three individuals from Cypress Hills (lower right) from the other two groups. One sample (unk) from Revelstoke, British Columbia was not photographed and as a result, we cannot confirm the subspecies.

Our PCA analysis with the complete dataset (103,268 SNP) showed the separation of *Z. l. pugetensis* and southern *Z. l. oriantha* from the other two groups (Figure 2A) along the first and second axis (1.70% and 1.13%, respectively). As *pugetensis* showed greater differentiation from the other three groups, the PCA was done without *pugetensis* and further separation was found (Figure 2B). The northern *oriantha* and *gambelii* separated from southern *oriantha* on PC1 (1.16%); and, three individuals, two *oriantha* and one *gambelii*, from Cypress Hills, a sky island in southeast Alberta, formed a third cluster separating on PC2 (1.08%). In both PCA analyses, the unknown individual from Revelstoke, British Columbia grouped with northern *oriantha* and *gambelii*. The result from our admixture analysis provides additional support for the divergence of *Z. l. pugetensis* from the rest, and some level of differentiation occurring among the remaining three subspecies (Figure S1).

**FIGURE 3:**
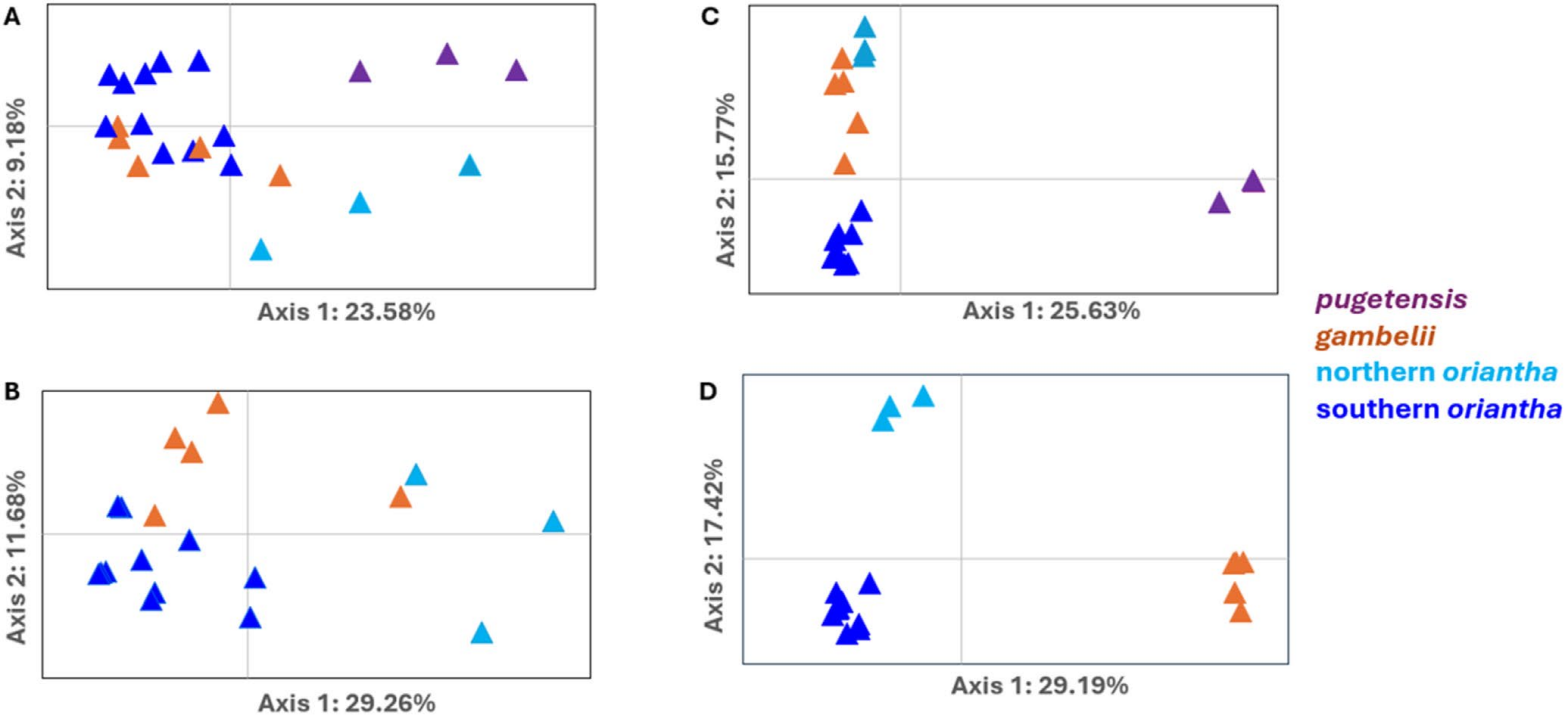
PCoA analyses for the four groups of *Zonotrichia* subspecies with outlier SNPs from the mitochondrial genome (A and B, 255 SNP) and Z chromosome (C and D, 580 SNP) with and without *pugetensis* (A and C, and B and D respectively). A. Clustering of the individuals based on mitochondrial genome all four groups included: *Gambelii* (orange), *pugetensis* (purple), southern *oriantha* (navy blue), and northern *oriantha* (teal blue). B. Clustering of the individuals based on mitochondrial genome excluding pugetensis samples. Clustering of the individuals based on Z chromosome SNPs, all four populations included (C) and excluding *pugetensis* (D).

**FIGURE 4:**
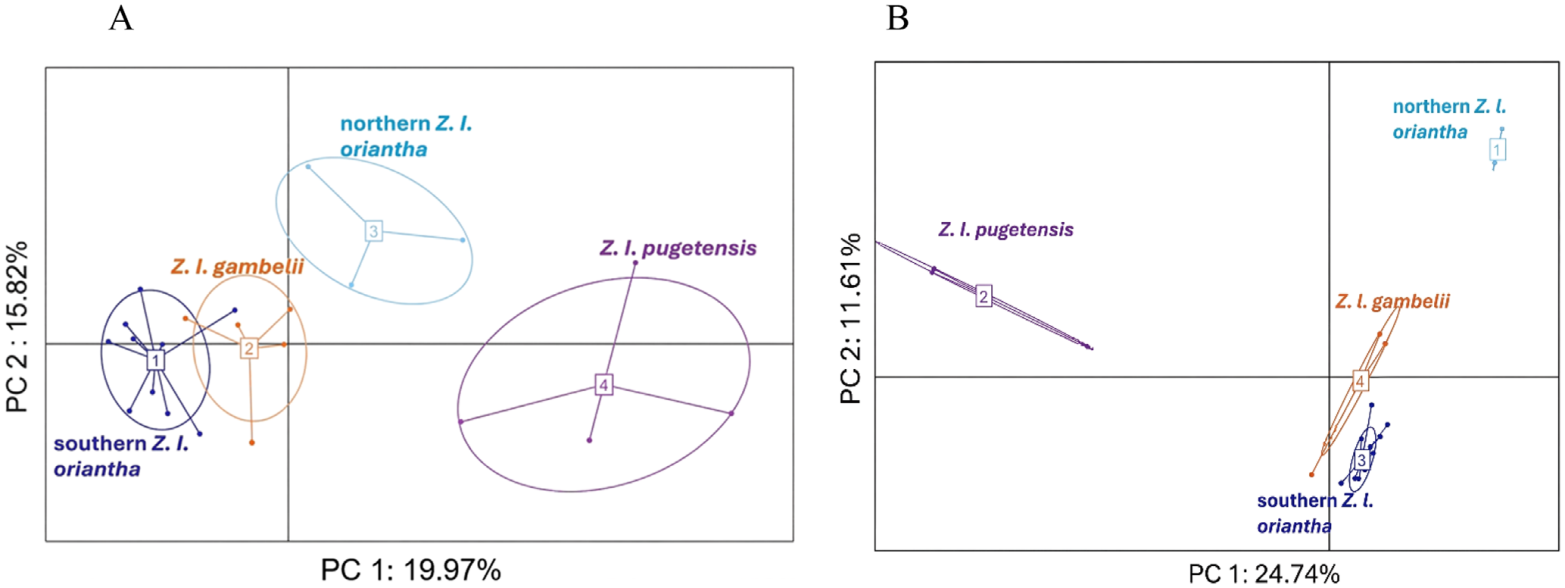
DAPC analysis of (A) 255 outlier SNPs in the mitogenome and (B) 580 outlier SNPs in the Z chromosome.

Using outlier SNPs from the mitochondrial (255 SNP) and the Z chromosome (580 SNP) datasets, PCoA results showed clustering corresponding to the subspecies and geography within *oriantha*. The mtDNA dataset showed loose clustering (Figure 3A,B). When all three subspecies are included (Figure 3A), *pugetensis* and northern *oriantha* separate from *gambelii* and southern *oriantha* on PCo1 (23.58%) and northern *oriantha* shows some separation on axis 2 (9.18%). When *pugetensis* is excluded, axis 1 (29.26%) separates northern *oriantha* from the other two groups (except one *gambelii*) and axis 2 (11.68%) separates some *gambelii* from southern *oriantha*, resulting in three loose clusters (Figure 3B). The Z chromosome dataset showed tighter clustering. PCo1 (25.63%) clearly separated *pugetensis* from the other subspecies and axis 2 (15.77%) helped separate the northern *oriantha* and *gambelii* from the other two groups. When *pugetensis* was removed, three clear groups were present: northern *oriantha*, *gambelii* and southern *oriantha* (Figure 3D, axis 1 29.19%, axis 2 17.42%). Separation of the four groups using DAPC was lower in the mtDNA dataset (PC1 19.97%, PC2 15.82%, Figure 4A) compared to the Z chromosome (PC1 24.74%, PC2 11.61%, Figure 4B), but both showed four groups and high separation of *pugetensis*. In the mtDNA dataset, *gambelii* and southern *oriantha* showed minimal overlap.

**FIGURE 5:**
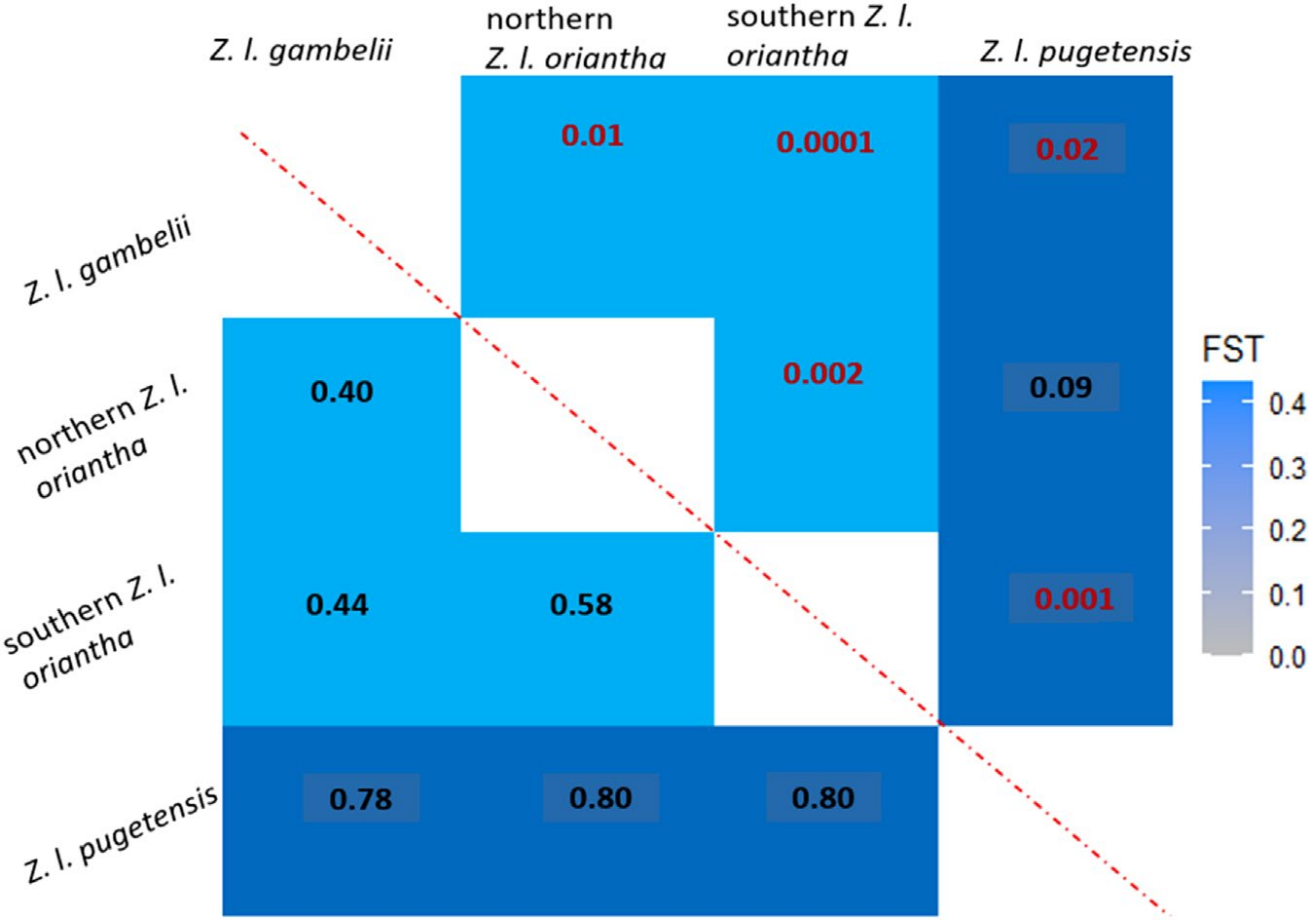
Pairwise *F*_ST_ values (below the diagonal) with the lcWGS data for the four groups (Z chromosome outlier SNPs) and associated *p* values (above the diagonal). Significant *p* values are in red.

Pairwise *F*_ST_ analyses (Figure 5) show statistically significant relationships (*p* < 0.05) which are similar to the PCoA for the whole genome dataset with all comparisons, except one. The exception is the *pugetensis*/northern *oriantha* pair; while the pairwise *F*_ST_ value for this pair is very high (0.80), the lack of significance (*p* = 0.09) may be due to a small sample size (*n* = 3 for each).

### 3.2 Genetic Differentiation Across the Whole Genome

**FIGURE 6:**
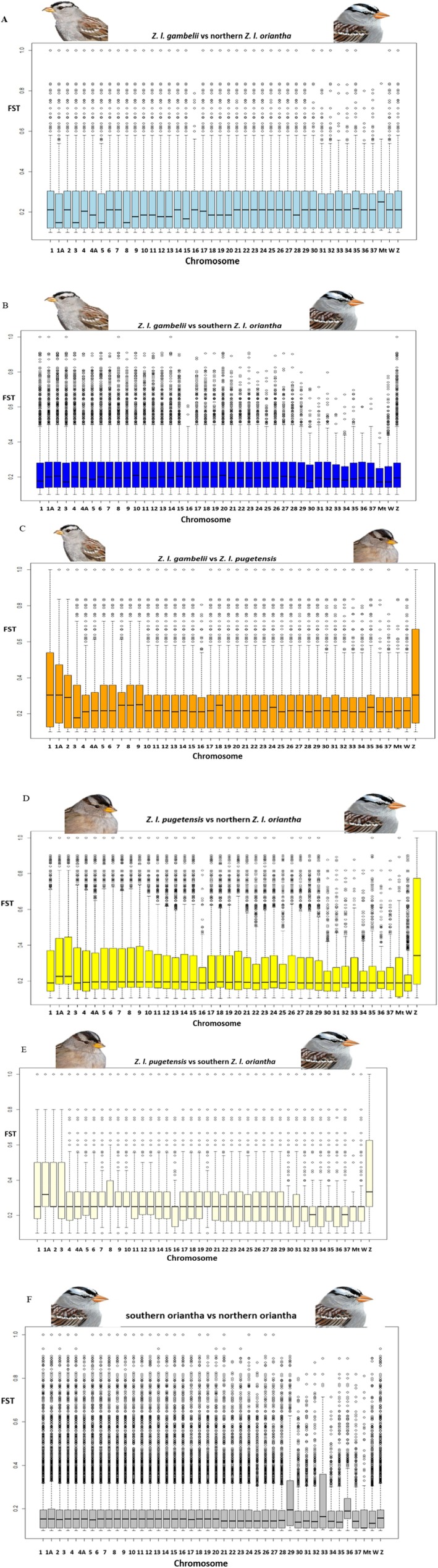
Boxplot of genetic differentiation (*F*_ST_) for comparison between the four populations. 42 chromosomes including mitochondrial, and the sex chromosomes Z and W were compared between the populations. (A) comparison between *Z. l. gambelii* and northern *Z. l. oriantha* shows that genetic differentiation is nearly uniform across the chromosomes except for a few chromosomes. (B) Between *Z. l. gambelii* and southern *Z. l. oriantha* genetic differentiation is lowest in the mitochondrial genome. (C) Comparison between *Z. l. gambelii* and *Z. l. pugetensis* shows differentiation to be highest in the Z chromosome followed by chromosomes 1, 1A and 2. (D) Genetic differentiation between *Z. l. pugetensis* and northern *Z. l. oriantha* is highest in the Z chromosome, followed by chromosomes 1A and 2, and relatively high in the mitogenome. (E) Genetic differentiation is highest in the Z chromosome for the *Z. l. pugetensis* and southern *Z. l. oriantha* pair. Differentiation is also high in chromosomes 1, 1A, 2 and 3. (F) Chromosomes 29 and 33 showed the highest value for genetic differentiation between the northern and southern *oriantha*.

*F*_ST_ was elevated on the Z chromosome across the majority of the subspecies pairs (Figure 6). For the comparison between *Z. l. gambelii* and northern *Z. l. oriantha*, *F*_ST_ values are more uniform across all chromosomes, except for very low values observed on chromosome 1A, 3, 5 and 8 and elevated values on the mitochondrial genome (Figure 6A). *F*_ST_ values for *Z. l. gambelii* and southern *Z. l. oriantha* are relatively uniform across all chromosomes, but noticeably lower for the mitochondrial genome, chromosomes 1, 3, and W (Figure 6B) relative to other chromosomes. Our results showed that when *Z. l. gambelii* was compared to *Z. l. pugetensis F*_ST_ values were elevated not only on the Z chromosome, but also on chromosomes 1, 1A, and 2; and lower on chromosome 3 (Figure 6C). For *Z. l. pugetensis* and northern *Z. l. oriantha F*_ST_ values are elevated for the Z chromosome, and chromosomes 1A and 2 (Figure 6D). *Z. l. pugetensis* and southern *Z. l. oriantha* comparisons show highly elevated *F*_ST_ values for the Z chromosome and chromosome 1A and lower on chromosomes 33 and 37 (Figure 6E). Lastly *F*_ST_ values are elevated for chromosomes 29, 33 and 36 and for the Z chromosome, but lower in the mitochondrial genome for the comparison between southern *Z. l. oriantha* and northern *Z. l. oriantha* (Figure 6F). Within pairs of subspecies, most consistently show higher or lower *F*_ST_ values on the Z chromosome and chromosomes 1, 1A and 2; and the values are higher for comparisons involving *Z. l. pugetensis* to all the other two subspecies, while lower *F*_ST_ values on either chromosome 1 or 1A for comparisons with *Z. l. gambelii*, and either southern or northern *Z. l. oriantha*.

### 3.3 Genes Potentially Linked to the Outlier SNPs

We identified 36 genes (Table S1) within 500 kb of our outlier loci (Figure S2), and produced a gene function table (Table 2) across all six comparisons. We noted two genes, LUZP2 and DYM, appeared in three of the comparisons, and MYO5B and CTIF appeared in two comparisons. The largest number of genes was identified in *gambelii*‐*pugetensis* (*n* = 13; 36%) followed by *gambelii*‐southern *oriantha* (*n* = 8; 22%); *pugetensis*‐northern *oriantha* (*n* = 8; 22%); *gambelii*‐northern *oriantha* (*n* = 6; 16%); and the lowest number of genes was found in *pugetensis*‐southern *oriantha* (*n* = 3; 8%) and southern *oriantha‐*northern *oriantha* (*n* = 2; 5%). Chromosome 1A and the Z chromosome contained the largest number of identified genes (8 and 15, respectively). Interestingly, the DYM gene linked to outlier loci on the Z chromosome was identified in three pairs of subspecies.

**TABLE 2:** Genes found around the outlier loci, function and occurrence in the subspecies. The genes marked with an asterisk appear in more than one pairwise comparison and those in bold appear in three comparisons.

Chr	Gene	Function/Ref	References	Subspecies combination
1A	SEMA3E	Positive regulation of cell migration	Sherman et al. 2022	gambelii_S.oriantha
	EXOC4	Involved in exocytosis	Sherman et al. 2022	gambelii_pugetensis
	SUV39H2	Cellular response to hypoxia	Sherman et al. 2022	pugetensis_N.oriantha
	FAM107B	Plays a role in spine formation	Mu et al. 2017	pugetensis_N.oriantha
	MEIG1	Essential for spermiogenesis	Sherman et al. 2022	pugetensis_N.oriantha
	DCLRE1C	Plays a role in DNA repair	Sherman et al. 2022	pugetensis_N.oriantha
	HSPA14	Heat shock protein family, in birds it protects cells during environmental stress	Feder and Hofmann 1999; Shehata et al. 2020; Sherman et al. 2022	pugetensis_N.oriantha
	DMTF1	Transcription regulation	Sherman et al. 2022	pugetensis_N.oriantha
3	TTC32	Protein binding	Sherman et al. 2022	gambelii_S.oriantha
4	FAM193A	unknown	Sherman et al. 2022	gambelii_S.oriantha
	RNF4	Transcription regulation	Sherman et al. 2022	gambelii_S.oriantha
5	LUZP2*	unknown		gambelii_pugetensis; pugetensis_N.oriantha; pugetensis_S.oriantha
7	KALRN	Plays a role in protein phosphorylation, locomotory behavior, social behavior	Sherman et al. 2022	gambelii_pugetensis
8	DAB1	Plays a role in neuron migration	Sherman et al. 2022	gambelii_pugetensis
	LRP8	Ventral spine cord development	Sherman et al. 2022	gambelii_pugetensis
22	KAT6A	Transcription regulation	Sherman et al. 2022	gambelii_pugetensis
	ANK1	Plays a role in cytoskeleton development	Sherman et al. 2022	pugetensis_S.oriatha
31	CACNA1F	Plays a role in vision, photoreceptor and skeletal muscle	An et al. 2015	pugetensis_S.oriantha
32	SLC25A11	Plays a role in mitochondrial metabolism	Sherman et al. 2022	S.oriantha_N.oriantha
35	STK19	Functions in DNA repair	van den Heuvel et al. 2024	gambelii_pugetensis
36	RNF31	May contribute to mechanisms of melanin internalization	Moreiras et al. 2022	gambelii_pugetensis
Z	DYM*	Golgi organization, plays a role in bone development, auditory function	Denais et al. 2011; Nagtegaal et al. 2012; Sherman et al. 2022	gambelii_pugetensis; gambelii_N.oriantha; pugetensis_S.oriantha
	MYO5B*	Actin cytoskeleton, microfilament motor activity, and auditory function	Nagtegaal et al. 2012; Sherman et al. 2022	gambelii_pugetensis; gambelii_N.oriantha
	HOOK3	Participates in protein transport	Sherman et al. 2022	gambelii_pugetensis
	GAK	Protein phosphorylation and golgi organization		gambelii_pugetensis
	PIGG	Plays a role in glycolipid biosynthesis	Sherman et al. 2022	gambelii_pugetensis
	ACAA2	Plays a role in cellular response to hypoxia, and auditory function	Nagtegaal et al. 2012	gambelii_N.oriantha
	SMAD7	Response to laminar fluid shear stress, auditory function.	Nagtegaal et al. 2012; Sherman et al. 2022	gambelii_N.oriantha
	CTIF*	Translation of mRNA molecules, auditory function	Nagtegaal et al. 2012; Sherman et al. 2022	gambelii_N.oriantha; S.oriantha_N.oriatha
	ZBTB7C	Transcription regulation	Sherman et al. 2022	gambelii_N.oriantha
	SMAD2	Regulates glucose response. In birds it plays a role in muscle development	Saneyasu et al. 2019; Sherman et al. 2022	gambelii_S.oriantha
	SKOR2	Transcription regulator	Sherman et al. 2022	gambelii_S.oriantha
	HDHD2	In birds it plays a role in response to environmental stress	Subba et al. 2026	gambelii_S.oriantha
	KATNAL2	In vertebrates, plays a role in pigmentation	Willsey et al. 2018	gambelii_S.oriantha
	ELAC1	Functions in tRNA repair	Sherman et al. 2022	pugetensis_N.oriantha
	ME2	Plays a role in mitochondrial function	Sherman et al. 2022	pugetensis_N.oriantha

## 4 Discussion

LcWGS data showed that the three subspecies are genetically distinct and *Z. l. oriantha* have a north–south split. While the complete genome dataset overall showed low support for genetic differences, higher resolution was found using outlier loci on the Z chromosome, and the mitochondrial genome provided lower resolution with some mixing of *Z. l. gambelii* and *Z. l. oriantha*.

Our study compared three of the five subspecies (*Z. l. gambelii, Z. l. oriantha, Z. l. pugetensis*) believed to have diverged in the last 18,000 years (Rand 1948) and the patterns we found using outlier loci support previous findings of Welke et al. (2021) and Taylor et al. (2021). Welke et al. (2021) used microsatellite data from 328 birds across their range and showed a genetic split among those three subspecies and within *Z. l. oriantha* separating northern (BC and AB) and southern (CO) populations. Our subspecies groups are similar to the PCA in Taylor et al. (2021) who showed separation of *oriantha* and *gambelii* using RADseq data, but not with their ADMIXTURE and maximum likelihood tree analyses. Their other analyses showed either two clusters with *oriantha*, *gambelii* and *leucophrys* (the three subspecies east of the Cascade and Coast Mountains) in both clusters or no monophyly for *oriantha* and *gambelii*. While the *oriantha* sampling sites from Taylor et al. (2021) are similar to ours (four samples listed in their appendix shows two from each of CO and AB), they had fewer *oriantha* samples and their *gambelii* samples were mostly from AK (9 of 10 samples) while ours were from BC, AB, CO and OR. Aside from differences in samples and the much larger number of samples in the microsatellite dataset, the lcWGS may provide higher resolution than RADseq and contribute to some of the differences in the patterns. Low‐coverage whole‐genome sequencing (lcWGS) provides genome‐wide representation making it well suited for population‐level inference, but this genome‐wide comes at the cost of increased genotype uncertainty at individual loci due to lower sequencing depth compared to RADseq (Widmayer et al. 2025; Lou et al. 2021). We also looked at outlier loci which the other studies did not, in part as either their dataset did not allow them to (Welke et al. 2021) or it was outside the scope of the paper (Taylor et al. 2021).

Focusing on differences with the mitogenome and Z chromosome outlier datasets, we see a similar pattern as the complete genome with *pugetensis* separating from the populations to the east (Figures 2, 3, 4). We get separation within the three groups east of the Rocky Mountains, however, with the mtDNA outlier loci we see some *gambelii* with higher affinities to each of the *oriantha* clusters than to the other *gambelii*. Sample sizes could be a factor; however, both of the previous studies also showed no differences in the mtDNA from these two subspecies. Evidence of intergradation has been reported between *Z. l. gambelii and Z. l. oriantha* especially along their contact zone in southwestern Alberta (Lein and Corbin 1990) and may explain the lower resolution with the mitochondrial data. It may also explain why our genomic data show the northern *oriantha* and *gambelii* as the two most genetically similar groups since many of the samples for those two groups are from Alberta. In contrast the Z chromosome outlier dataset showed clear separation of all four groups though separation among the eastern populations was more apparent when *pugetensis* was removed (Figures 3 and 4).

The divergence within the *Zonotrichia leucophrys* clade is linked to the historical processes associated with range expansions at the end of the Pleistocene (Zink et al. 1991; Morton 2002). Taylor et al. (2021) and Welke et al. (2021) pointed to divergence especially for western *nuttalli* and *pugetensis* separating from *gambelii, oriantha* and *leucophrys* with a combination of microsatellite and nuclear SNP markers corresponding to isolation in refugia on either side of the Rocky Mountains. However, there was no clear pattern to divergence among *Z. l. gambelii*, *Z. l. oriantha* and *Z. l. leucophrys* in Taylor et al. (2021) and aside from the PCA, their results show evidence of the three subspecies mixing. Our data support the isolation of *pugetensis* from both *gambelii* and *oriantha* likely being the result of geographic separation between *pugetensis* and the other two subspecies, and the high fidelity to their breeding sites (Morton 2002), but it also showed differences among *gambelii* and *oriantha*. The genetic structuring we observe between *gambelii* and *oriantha* despite ongoing gene flow suggests that even shallow geographical and ecological barriers can maintain incipient divergence.

Within *oriantha*, populations from the northern part of the range (Alberta and British Columbia) clustered separately from those in the south (Colorado). Welke et al. (2021) had previously documented this separation with microsatellite data using a larger number of samples. High fidelity of *oriantha* populations in Colorado, divergence time and geographic distance or the sampling gap may also factor into the genetic divergence between the two *oriantha* groups. We also saw separation of three birds (two northern *oriantha* and one *gambelii*) from Cypress Hills. Cypress Hills is a sky island in southeastern Alberta and southwestern Saskatchewan known to contain genetically distinct populations of several species (Haché et al. 2017; Dempsey et al. 2020; Carpenter et al. 2022) and contains habitat more similar to the Rocky Mountains than the surrounding prairie habitat. Additional sampling across the range is needed to better understand the separation within *Z. l. oriantha* populations using lcWGS data and the factors that have led to their divergence.

### 4.1 Genomic Differences

When examining all 42 chromosomes (Figure 6), we found elevated genetic differentiation in all comparisons involving *pugetensis*, and between northern and southern *Z. l. oriantha*, particularly on the Z chromosome. As the Z chromosome has a smaller effective population size, it is more sensitive to drift and may also be subject to sex‐linked selection dynamics (Charlesworth et al. 1987; Oyler‐McCance et al. 2015). McCallum et al. (2024) suggested that recurrent selection, including diversifying selection, has shaped divergence on the Z chromosome in *Z. atricapilla* and *Z. leucophrys* , particularly between *pugetensis* and *gambelii*.

A large block of elevated differentiation on chromosome 1A was also reported in the McCallum et al. (2024) study and hypothesized to result from reduced recombination and/or selection maintaining linkage among co‐adapted loci. We observed similar patterns when we mapped them to the zebra finch genome: all comparisons involving *pugetensis* showed elevated differentiation on chromosome 1A, whereas *oriantha–gambelii* comparisons did not. In other avian taxa, including warblers and robins, high differentiation on chromosome 1A has been attributed to mitonuclear coadaptation, as this chromosome is enriched for nuclear‐encoded mitochondrial genes (Morales et al. 2018; Wang et al. 2021).

Our mitochondrial genome analyses mapped to zebra finch genome revealed relatively low differentiation between northern and southern *oriantha* compared to the Z chromosome and autosomal loci. One possible explanation is historical mitochondrial introgression or positive selection maintaining shared haplotypes across geographically structured nuclear backgrounds (Bazin et al. 2006; Seixas et al. 2018; Pereira et al. 2021). While female‐biased dispersal could reduce mitochondrial divergence, this is unlikely to fully explain the pattern as previous microsatellite data showed clear population structure (Welke et al. 2021), suggesting limited recent gene flow.

In contrast, mitochondrial differentiation between *gambelii* and both *oriantha* groups was higher than expected under a scenario of mtDNA capture through past hybridization, which typically reduces divergence (Toews and Brelsford 2012). Instead, elevated *F*_ST_ at mitochondrial outlier loci may reflect lineage‐specific adaptation to local environments, such as climate, consistent with mitonuclear coadaptation (Dowling et al. 2008; Morales et al. 2018; Bar‐Yaacov et al. 2015). This hypothesis is supported by the lack of corresponding nuclear differentiation, implying selection rather than drift or neutral gene flow is driving mitochondrial divergence.

### 4.2 Genes Associated With Regions of Differentiation

Our study identified 36 genes within 500 kb of our outlier loci across the genome; four of these genes were found in more than one subspecies combinations (LUZP, DYM, MYO5B, and CTIF). Some genes, such as DYM, FAM107, LRP8, CACNA1F, and SMAD2, have roles in bone formation and skeletal muscle development (Denais et al. 2011; Nagtegaal et al. 2012; Mu et al. 2017; Sherman et al. 2022), which may be important for locomotion in animals. In addition to DYM's gene role in bone and muscle development, it is reported to be involved in sound processing in mice, along with other genes such as CTIF, ACAA2, SMAD7, and MYO5B (Nagtegaal et al. 2012). RNF31 and KATNAL2 are reported to play a role in melanin internalization and pigmentation in humans and mice (Moreiras et al. 2022; Willsey et al. 2018). These genes may be relevant in the divergence of bill coloration and lore pigmentation in our study populations. Further, HDHD2, HSPA14, CTIF, and KALRN were found to be linked to other important functions, such as response to environmental stress, auditory function, and locomotory and social behavior, respectively (Sherman et al. 2022; Shehata et al. 2020; Nagtegaal et al. 2012). These genes may also contribute to the migratory behavior and adaptation to local environment in our study populations, such as *gambelii*, that are highly migratory. With the identification of two genes linked to pigmentation within the regions showing elevated differentiation, it would be interesting to see if these correspond to variation in the bill and lore colouration, which are two major phenotypic features used to differentiate these subspecies. It will also be informative to know if the genes linked to stress, hypoxia, skeletal, and muscle development contribute to migration in this population.

## 5 Conclusions

By integrating low‐coverage whole‐genome sequencing with mitochondrial and Z chromosome analyses, this study clarifies patterns of recent divergence in the white‐crowned sparrow ( *Zonotrichia leucophrys* ). Across all analyses, *Z. l. pugetensis* was consistently the most genetically differentiated lineage, whereas divergence among the Rocky Mountain subspecies was more complex. We identified a clear north–south split within *Z. l. oriantha*, with northern populations showing greater genetic similarity to *Z. l. gambelii* than to southern *oriantha*, consistent with recent divergence, incomplete lineage sorting, and localized gene flow.

Genetic differentiation was highly heterogeneous across the genome, with the strongest divergence concentrated on the Z chromosome, highlighting the disproportionate role of sex‐linked loci in structuring variation among closely related subspecies. In contrast, mitochondrial markers showed lower resolution and evidence of mitonuclear discordance, underscoring the importance of jointly analyzing multiple genomic compartments. Regions of elevated differentiation were associated with genes linked to pigmentation, skeletal and muscle development, auditory function, and environmental stress responses, suggesting that selection on a limited number of loci may contribute to phenotypic divergence.

Overall, our results demonstrate that substantial genetic structure can emerge in the absence of strong physical barriers, driven by a combination of sex‐linked selection, local adaptation, and historical demography. This work highlights the utility of lcWGS for resolving shallow divergence and provides a foundation for future studies linking genomic differentiation to phenotypic and ecological variation in widespread songbirds.

## Author Contributions

**Patricia B. Osagie:** formal analysis (lead), funding acquisition (supporting), investigation (equal), methodology (equal), writing – original draft (equal), writing – review and editing (equal). **Juan Enciso‐Romero:** formal analysis (supporting), methodology (supporting), software (lead), writing – review and editing (equal). **Theresa M. Burg:** conceptualization (lead), funding acquisition (lead), project administration (lead), resources (lead), supervision (lead), writing – original draft (supporting), writing – review and editing (lead).

## Funding

This work was supported by the Natural Sciences and Engineering Research Council of Canada and Alberta Conservation Association.

## Conflicts of Interest

The authors declare no conflicts of interest.

## Supporting information


**Table S1:** Gene occurrence across subspecies pairs, providing an overview of genes that are common to each pair.
**Figure S1:** NGSAdmix plot shows support for the divergence of *Z. l. pugetensis* from other subspecies and some level of differentiation for the other three groups at *K* = 3 and *K* = 4.
**Figure S2:** *F*_ST_ scans depicting regions that may be contributing to divergence of the four groups as determined by peaks and or high values based on the lcWGS dataset. The red line is the 99.9% threshold set for the identification of the outlier SNPs. SNPs/genes above the line are considered outliers. Chromosomes are arranged in the order from left chr. 1, 1A … 28, Z, 29, 30, W, 31…37.

## Data Availability

The dataset for this work is archived in The Federated Research Data Repository. https://doi.org/10.20383/103.01131.
