# Supplementary material for: Genomic and Mitonuclear Patterns of Divergence Among Recently Diverged White‐Crowned Sparrow Subspecies
Source: Ecol Evol. 2026 May 14;16(5):e73651. doi: 10.1002/ece3.73651 (PMC13175713; doi:10.1002/ece3.73651)
Supplement: Supplementary file 1 — Table S1: Gene occurrence across subspecies pairs, providing an overview of genes that are common to each pair. Figure S1: NGSAdmix plot shows support for the divergence of Z. l. pugetensis from other subspecies and some level of differentiation for the other three groups at K = 3 and K = 4. Figure S2: F ST scans depicting regions that may be contributing to divergence of the four groups as determined by peaks and or high values based on the lcWGS dataset. The red line is the 99.9% threshold set for the identification of the outlier SNPs. SNPs/genes above the line are considered outliers. Chromosomes are arranged in the order from left chr. 1, 1A … 28, Z, 29, 30, W, 31…37. [file ECE3-16-e73651-s001.docx]

**Supplementary data**

**Table S1.** Gene occurrence across subspecies pairs, providing an overview of genes that are common to each pair.

| **Chromosome** | **gambel vs Puget** | **gambel vs N. oriant** | **gambel vs S. oriant** | **puget vs N. oriant** | **Puget vs S. oriant** | **S. orient vs N. oriant** |
| --- | --- | --- | --- | --- | --- | --- |
| 1A |  |  |  |  |  |  |
|  | EXOC4 |  | SEMA3E | SUV39H2 |  |  |
|  |  |  |  | FAM107B |  |  |
|  |  |  |  | MEIG1 |  |  |
|  |  |  |  | DCLRE1C |  |  |
|  |  |  |  | HSPA14 |  |  |
|  |  |  |  | DMTF1 |  |  |
| 3 |  |  | TTC32 |  |  |  |
| 4 |  |  | FAM193A  RNF4 |  |  |  |
| 5 | **LUZP2*** |  |  | **LUZP2*** | **LUZP2*** |  |
| 7 | KALRN |  |  |  |  |  |
| 8 | DAB1  LRP8 |  |  |  |  |  |
| 22 | KAT6A |  |  |  | ANK1 |  |
| Z | **DYM***  MYO5B*  HOOK3  GAK  PIGG | ACAA2  MYO5B*  **DYM***  SMAD7  CTIF*  ZBTB7C | SMAD2  SKOR2  HDHD2  KATNAL2 | ELAC1  ME2 | **DYM*** | CTIF* |
| 31 |  |  |  |  | CACNA1F |  |
| 32 |  |  |  |  |  | SLC25A11 |
| 35 | STK19 |  |  |  |  |  |
| 36 | RNF31 |  |  |  |  |  |


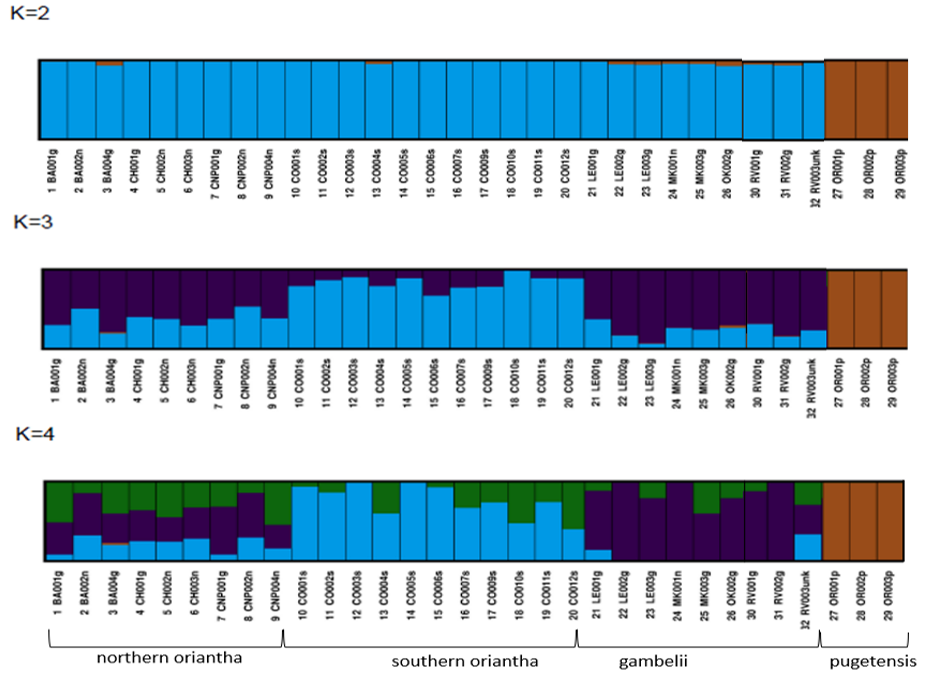


**Figure S1**: NGSAdmix plot shows support for the divergence of *Z. l. pugetensis* from other subspecies and some level of differentiation for the other three groups at K=3 and K=4.


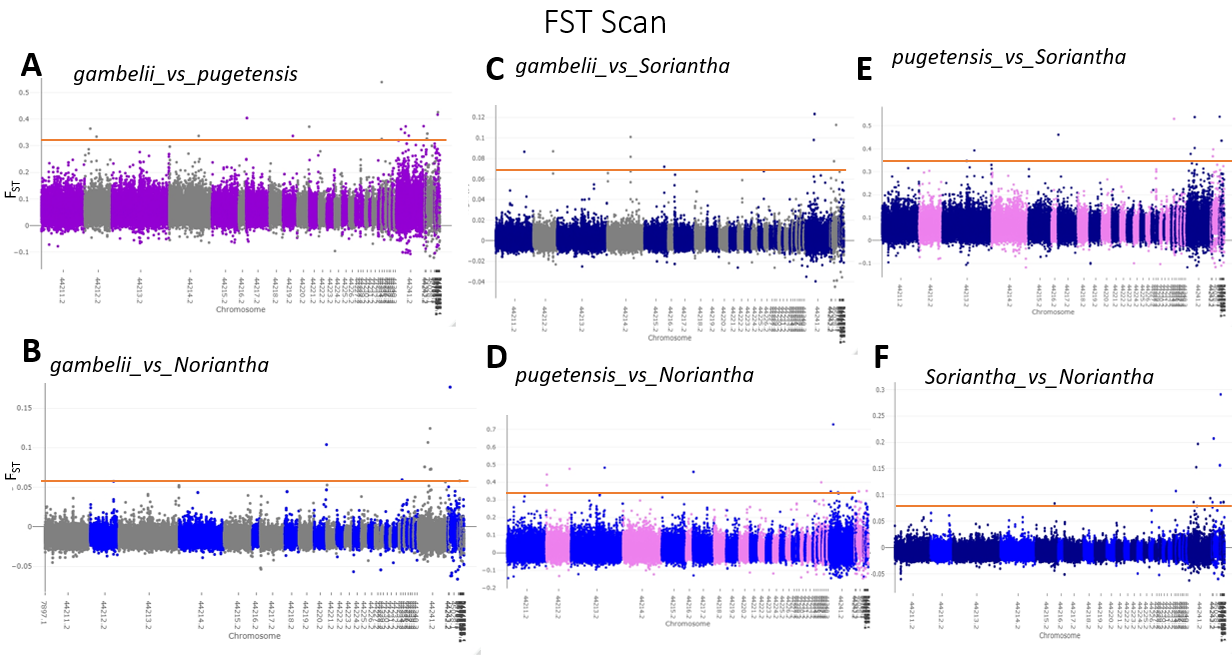


**Figure S2**: F_ST_ scans depicting regions that may be contributing to divergence of the four groups as determined by peaks and or high values based on the lcWGS dataset. The red line is the 99.9% threshold set for the identification of the outlier SNPs. SNPs/genes above the line are considered outliers. Chromosomes are arranged in the order from left chr. 1, 1A…28, Z, 29, 30, W, 31…37
